# Genomic characterization of the attenuated human cytomegalovirus strain TR-VAC developed for subviral particle vaccine production

**DOI:** 10.1128/mra.00397-26

**Published:** 2026-09-10

**Authors:** Hanno Schmidt, Charlotte Hewel, Nicole Büscher, Matthias Linke, Bodo Plachter

**Affiliations:** 1 Institute of Virology, University Medical Center of the Johannes Gutenberg-University Mainz 39068, Mainz, Germany; 2 Institute of Human Genetics, University Medical Center of the Johannes Gutenberg University Mainz 39068, Mainz, Germany; Katholieke Universiteit Leuven, Leuven, Belgium

**Keywords:** Human cytomegalovirus, genome sequencing, vaccine candidate, genomic validation

## Abstract

We report the complete genome sequence of the attenuated human cytomegalovirus strain TR-VAC, developed for subviral particle vaccine production. Oxford Nanopore duplex sequencing confirmed all engineered modifications, including UL130 repair, UL25 stop codons, ddFKBP insertion, GFP deletion, and retention of the bacterial artificial chromosome backbone, without large-scale structural rearrangements.

## ANNOUNCEMENT

Human cytomegalovirus (HCMV, Orthoherpesviridae) is a major cause of congenital disease and of complications in transplant recipients (1). A genetically attenuated HCMV-strain, termed TR-VAC, was recently developed as a seed virus for the production of a vaccine, based on subviral particles termed Dense Bodies (2). Key features are restoration of pentameric complex expression, abrogation of UL25 expression, and conditional regulation of IE1/IE2. While the genetic design and functional properties of TR-VAC have been described (2), a complete genome sequence has not been reported. Here, we provide the full-length genome sequence of TR-VAC and confirm the integrity of all engineered modifications at nucleotide resolution. This resource establishes a publicly accessible reference sequence for the genetically attenuated seed strain and provides a basis for quality control, reproducibility, and further preclinical or clinical development of dense body-based HCMV vaccine platforms.

TR-VAC was constructed based on the laboratory strain Towne (3, 4). The TR-VAC genome was propagated as bacterial artificial chromosome (BAC) in *Escherichia coli* SW102 grown in lysogeny broth medium with chloramphenicol at 32°C and purified using the NucleoBond PC 500 kit (Macherey-Nagel) following the manufacturer’s protocol. Purified BAC DNA was quantified on a Qubit 2.0 fluorometer (Invitrogen), its quality was assessed using a NanoDrop 2000 spectrophotometer (ThermoFisher), and it was linearized by digestion with restriction enzyme PmeI.

A genomic DNA library was prepared using Oxford Nanopore Technologies (ONT) ligation sequencing kit V14 (SQK-LSK114) according to the manufacturer’s instructions and sequenced on a PromethION24 device (ONT) using an R10.4.1 flow cell (FLO-PRO114M). Duplex base-calling was performed using Dorado v0.3.0 (ONT). Only duplex reads were aligned to the HCMV Towne-BAC reference genome KF493877.1 using epi2me-labs/wf-alignment v0.5.0 (ONT). A consensus sequence was generated by samtools consensus v1.16.1 (5) and aligned to the HCMV Merlin reference genome NC_006273.2 using nucmer from the MUMmer4 package (6) and linearized relative to the reference.

**Fig 1:**
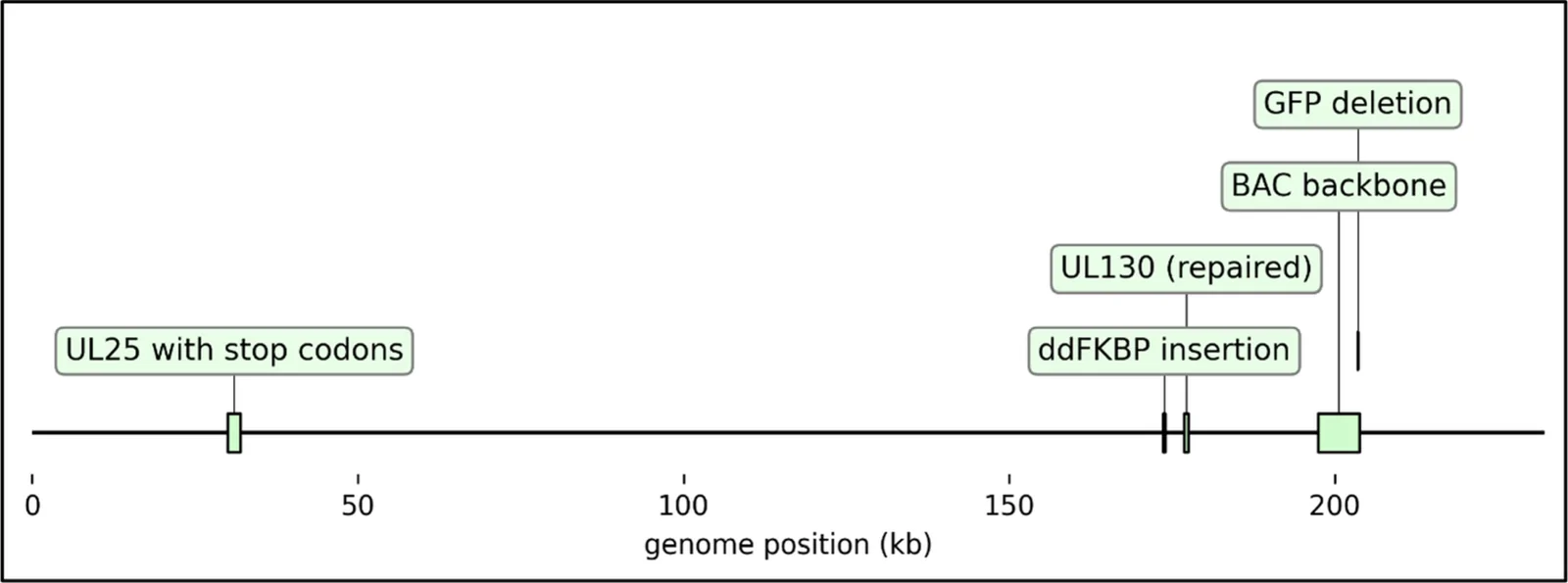
Schematic representation of the TR-VAC genome and engineered modifications. All engineered loci are shown on the fully reconstructed genome linearized according to the Merlin reference. Each locus was manually inspected and verified against the intended design. The genomic configuration is the same as described by Krauter et al. (2) and illustrated in Fig. 1 therein.

Sequencing yielded 8,829 reads with an N50 of 23,347 bp. Of those, 1,678 duplex reads were used for reference-guided consensus generation, producing a 232,183 bp gap-free genome sequence with 116× coverage. Whole-genome alignment inspection and dotplot analysis revealed no large-scale structural rearrangements relative to the Merlin reference (7). The presence of HCMV-expected genes was confirmed using VAPiD v1.6.7 (8). The presence of the FKBP-derived destabilization domain at the engineered insertion locus was confirmed by BLASTN searches and read mapping inspection (Fig. 1; Table 1). The UL130 sequence of the assembled genome lacked the double adenine insertion near the C-terminus that is characteristic of the nonfunctional Towne UL130 allele (9). Consistently, translation of the open reading frame showed a Merlin-like C-terminal amino acid sequence rather than the modified Towne variant, confirming the intended repair. In UL25, we confirmed all 16 ATG codons being changed, with 11 of them changed to TGA stop codons. The reconstructed viral genome includes approximately 6 kb of BAC backbone sequence originating from the cloning vector. The deletion of green fluorescent protein (GFP) was confirmed by manual inspection and BLASTN searches. Collectively, the sequence analyses confirmed that all expected engineered modifications were present in the TR-VAC genome.

**TABLE 1:** Genomic features and sequence modifications^*a*^

Feature	Start position	End position	Notes
BAC backbone	197464	203815	6,351 bp BAC
GFP deletion	203547	203548	1,550 bp deleted
ddFKBP insertion	173660	173989	329 bp inserted
UL130 repair	176884	177529	C-terminus Merlin-like
UL25 ATG substitution	30055	30057	ATG > TTG
UL25 ATG substitution	30146	30148	ATG > TTG
UL25 ATG substitution	30214	30216	ATG > TTG
UL25 ATG substitution	30572	30574	ATG > TGA (stop)
UL25 ATG substitution	30618	30620	ATG > GAA
UL25 ATG substitution	30744	30746	ATG > GAA
UL25 ATG substitution	30754	30756	ATG > TGA (stop)
UL25 ATG substitution	30895	30897	ATG > TGA (stop)
UL25 ATG substitution	31231	31233	ATG > TGA (stop)
UL25 ATG substitution	31260	31262	ATG > TGA (stop)
UL25 ATG substitution	31312	31314	ATG > TGA (stop)
UL25 ATG substitution	31403	31405	ATG > TGA (stop)
UL25 ATG substitution	31663	31665	ATG > TGA (stop)
UL25 ATG substitution	31734	31736	ATG > TGA (stop)
UL25 ATG substitution	31870	31872	ATG > TGA (stop)
UL25 ATG substitution	31888	31890	ATG > TGA (stop)

^*a*^ All listed features and modifications were confirmed by manual inspection of read mappings, genome alignments, BLAST searches, or a combination thereof. Coordinates refer to the submitted TR-VAC genome sequence, oriented and linearized relative to the HCMV Merlin reference (NC_006273).

## Data Availability

All sequencing data are available under National Center for Biotechnology Information BioProject PRJNA1426965 with Sequence Read Archive accession number SRR37325747. The assembled and annotated genome has been deposited in GenBank under accession number PZ137009.
